# Evaluation of the Disinfection Capacity of Electrolyzed Water and Growth Rates of *Listeria monocytogenes* on Chives at Different Storages for Ensuring Microbiological Quality of Fresh Vegetable Foods

**DOI:** 10.3390/foods15050957

**Published:** 2026-03-09

**Authors:** Hyeongmo An, Hyeja Chang

**Affiliations:** Department of Food and Nutrition, Dankook University, Cheonan 31116, Republic of Korea; 32182539@dankook.ac.kr

**Keywords:** disinfection method, electrolyzed water, growth rate of *Listeria monocytogenes*, fresh-cut vegetable

## Abstract

Fresh vegetables, especially green onions and chives, are raw ingredients widely consumed by Koreans, and have been linked to *Listeria monocytogenes*-induced food poisoning. This study aimed to assess microbial contamination levels in commercially available fresh-cut vegetables and produce, compare the effects of different types and concentrations of disinfectants on green onions and chives, and determine the growth rate of *L*. *monocytogenes* on chives under different storage conditions. Among the five fresh-cut vegetable mix salad products, the average total mesophilic count (TMC) was 2.00 log CFU/g, whereas the crown daisies exhibited the highest levels of raw produce contamination (TMC of 4.14 log CFU/g). The disinfection experiments indicated the elevated disinfectant capacities of electrolyzed water as well as washing under running water against *Escherichia coli* and *L*. *monocytogenes*. Enhanced anti-TMC ability of electrolyzed water were observed in acidic 30 ppm (pH 3.2) and 60 ppm (pH 5.6) of HOCl, and alkaline 100 ppm (pH 8.1) and 200 ppm (pH 8.8) of NaClO. Moreover, in the *L. monocytogenes* inoculation experiment in chive, the growth rates at 5 °C, 12 °C, and 30 °C were −0.002, 0.023, and 0.030 log CFU/g/h, respectively. This observation suggests that *L. monocytogenes* cannot grow on chives if stored at 5 °C but can at 12 °C. This study provides scientific evidence to guide the management of microbial quality of fresh produce and fresh-cut vegetables for safer meal provision in home and eating-out settings.

## 1. Introduction

Green onions and chives are representative vegetables commonly consumed as raw-vegetable dishes in Korea, and fresh-cut vegetables are widely used for health benefit dishes at childcare, school, and elderly care foodservice units [1]. Even when refrigerated, these raw-vegetable dishes can be contaminated with *Listeria monocytogenes* or *Escherichia coli*, resulting in a higher risk of food poisoning [2,3]. Nevertheless, only a few studies [4,5] have investigated the microbiological contamination levels of fresh-cut vegetables or salad dishes in Korea. Evidence regarding control methods for ensuring the microbial quality of fresh-cut vegetables and produce, in terms of the prevalence of *L*. *monocytogenes*, user-friendly disinfection methods, and a growth model of *L. monocytogenes* by storage temperatures, is very limited [6,7].

According to the Centers for Disease Control and Prevention (CDC), *E*. *coli* O157:H7 and *L. monocytogenes* were the main causative pathogens of fresh produce related food poisoning from 2011 to 2021 [8]. In 2020, foodborne illness occurred in 35 patients due to the consumption of vegetables contaminated with *L. monocytogenes*. Six pathogens: norovirus, *Campylobacter*, *Salmonella*, *Clostridium perfringens*, *L. monocytogenes*, and Shiga toxin-producing *E. coli*, have been estimated to cause 9 million cases of acquired foodborne illnesses and 53,300 hospitalizations in the United States [2]. Especially, *L. monocytogenes* is responsible for outbreaks associated with the consumption of celery, and has been isolated from fresh produce such as spinach, lettuce and cucumbers [9]. But in Korea, studies on the prevalence of *L. monocytogenes* and *E. coli* on fresh-cut vegetables and fresh vegetables, especially those from highly contaminated soil, are limited.

Moreover, to ensure the microbial safety of unheated raw-vegetable-based dishes, disinfection is crucial during food processing. The Ministry of Food and Drug Safety in Korea (KMFDS) allows the use of seven types of disinfectants for the reduction of pathogenic microorganisms from foods [10,11]. Among them, hypochlorous acid (HOCl) and sodium hypochlorite (NaOCl) have been widely used in foodservice settings and are classified as chlorine-based disinfectants. HOCl exhibits higher antibacterial properties against fungi and bacteria. But, at certain concentrations above the standards, HOCl can cause chemical burns. Similarly, NaOCl, an important ingredient of household bleach, can cause skin irritation, edema symptoms, and inflammatory reaction. For minimizing such risks, the KMFDA suggests that disinfectants must be used at the concentration specified by the manufacturer, and their residues must be removed from the final food items by washing them with potable water [10,11]. But the process is time-consuming and prone to inaccuracies when implemented in institutional foodservice or eating-out facilities. A device that generates electrolyzed water enables faster, more accurate, and easier sanitization; however, its use in foodservice facilities remains limited because of financial restrictions. Moreover, empirical research evaluating the disinfecting capacity of such electrolyzed water when used with fresh produce and fresh-cut vegetables is lacking.

In addition, the storage temperature of fresh salad dishes during transportation affects their microbiological quality before consumption. Previous studies have reported the growth rates of *L. monocytogenes* in onion, cabbage [4], and lettuce [12,13] under storage conditions. However, the growth rate of *L. monocytogenes* in chive, which are favorite in Korea and are frequently consumed, has not been researched. Moreover, in Korea, 10 °C is the legally permitted storage temperature, according to the Korea Food Code [14], but the possibility of abuse by the cold chain system still exists. In addition, no research has tried to demonstrate how the growth of *L. monocytogenes* in chives is affected by improper temperature control during distribution. Therefore, the objectives of this study are (1) to assess the microbial contamination levels in commercially available fresh-cut vegetables and produce; (2) to compare the effects of different types and concentrations of disinfectants on green onions and chives, which are widely consumed with non-heated dishes in Korea; and (3) to determine the growth rate of *L. monocytogenes* on chives under different distribution conditions.

## 2. Materials and Methods

### 2.1. Microbiological Quality of Fresh-Cut Vegetables and Produce

#### 2.1.1. Sample Preparation

The four types of produce samples used in the present study were cucumbers, lettuce, crown daisy, and carrots, which were purchased at a traditional market in Seoul from September 2024 to March 2025. As shown in Table 1, five types of fresh-cut vegetable salad products were purchased online and delivered by a large-scale grocery store. All samples were analyzed immediately after procurement. For microbial tests, produce samples were briefly washed once under running water, whereas fresh-cut vegetables were used without washing, as indicated on the product labels. The temperature of running water was measured with a K-type thermometer (SK Sato, Tokyo, Japan) and was 24 ± 0.15 °C. The water flow velocity of the preliminary washing was 0.45 m/s, measured using the bucket method. All experiments were performed in duplicates.

#### 2.1.2. Microbial Analyses

Microbiological analyses were performed in accordance with the Korean Food Code [14], as suggested by the KMFDS. Quantitative experiments were conducted to assess the levels of total mesophilic count (TMC), *E. coli*, and *L. monocytogenes*. Each sample, weighing 25 g was placed in a sterilized stomach bag (Nasco, Inc., Fort Atkinson, WI, USA) containing 225 mL of 0.1% peptone water and homogenized for 2 min using a stomacher. Tenfold serial dilutions from 10^−1^ to 10^−4^ were prepared. Subsequently, 1 mL of the diluted suspension was placed on Petri dishes of plate count agar (PCA), eosin methylene blue (EMB) agar, and Oxford agar, and incubated at 37 °C for 48 h. Subsequently, the levels of TMC, *E. coli*, and *L. monocytogenes* were measured. Each analysis was performed in duplicates.

##### TMC

To measure the TMC, 25 g of the sample was processed as described above, and 1 mL of the diluted sample was aseptically dispensed into sterile Petri dishes. Subsequently, 15 mL of PCA (KisanBio, Seoul, Republic of Korea) maintained at 50 °C was poured into the Petri dishes containing the sample, mixed, and incubated at 36.5 °C for 48 h to count the colonies.

##### Presumptive *E. coli*

For *E. coli* counts, 25 g of the sample was processed as described above. Then, 1 mL of the diluted sample solution was dispensed onto sterile Petri dishes, 15 mL of EMB agar (KisanBio) was poured into the plate and mixed, and the plate was incubated at 36.5 °C for 48 h. *E. coli* was verified based on the findings of Leininger et al.’ [15], who used EMB agar to differentiate *E. coli* from gram-negative mastitis pathogens. Colonies with a green metallic sheen [15,16] were counted as *E. coli*. The confirmatory IMViC test-Indole test, Methyl red test, Voges-Proskauer test, and Citrate test-was not performed.

##### Presumptive *L. monocytogenes*

For the quantitative analysis of *L. monocytogenes*, the homogenization and dilution processes described above were applied. Then, 1 mL of the diluted sample solution was dispensed onto sterile Petri dishes, 15 mL of Oxford agar (KisanBio),was poured and mixed, and then the plates were incubated at 36.5 °C for 48 h. Gray-green colonies surrounded by a black halo on the selective isolation medium [14,16,17], Oxford agar, were counted. No confirmation test (i.e., homolysis or phosphatidylinositol phospholipase test) was conducted.

### 2.2. Comparison of the Disinfection Efficacy of Different Disinfectant Types

A total of seven conditions were established to evaluate disinfecting power on green onions and chives. The sample that was not washed with water was used as the control group. The other six were briefly washed once and used as experimental groups: a sample washed three times with water; a sample disinfected with 100 ppm NaClO prepared using a commercially available chemical disinfectant (Yuhan Clorox, Seoul, Republic of Korea); and the remaining four samples were treated with four types of electrolyzed water—acidic 30 ppm HOCl (pH 3.2), acidic 60 ppm HOCl (pH 5.6), alkaline 100 ppm NaClO (pH 8.1), and alkaline 200 ppm NaCIO (pH 8.8)—produced with a hybrid-type disinfectant water manufacturing device (FF&E Co., Seoul, Republic of Korea). Each sample was immersed in the set condition for 5 min and then immersed in 1 L of sterilized distilled water at an ambient temperature of 19 °C for 30 s to remove any residual chlorine before the microbial tests, in accordance with the standards of the Ministry of Food and Drug Safety Act [10,11]. Then, the microbiological analyses were done to quantify TMC, *E. coli*, and *L. monocytogenes*.

### 2.3. Growth Rate of L. monocytogenes Inoculated on Chives

The strain used in the inoculation experiments was *L. monocytogenes* ATCC 15313, which was purchased from the Korean Culture Center of Microorganisms. The strain was received in a freeze-dried state. It was suspended in sterilized saline solution and cultured in tryptic soy broth at 30 °C for 24 h. After the process was repeated twice, the final culture solution was used to inoculate 25 g of chive samples, respectively, indicating an inoculation load of 3–4 log CFU/mL for the growth experiments. The inoculated samples were packaged in Whirl-Pak bags (Nasco) by manually pushing the air out without any special equipment, and then stored at 5 °C, 12 °C, and 30 °C for 0, 1, 3, and 5 days. The reasons for setting these three experiment temperatures were as follows: 5 °C is the refrigeration temperature of the HACCP system, 12 °C is the non-compliant temperature that exceeds the distribution standard of 10 °C set by the Korea Food Code, and 30 °C is the average ambient temperature. At the end of each storage duration, the samples were removed from their packaging and used for the analysis of *L. monocytogenes* growth.

For the development and validation of the predictive growth models, the Baranyi and Roberts function [18] was applied to estimate the growth behavior of *L. monocytogenes* during storage and to construct primary growth curves. The key model parameters were estimated using the ComBase browser (https://combase.errc.ars.usda.gov/): maximum specific growth rate (µ), lag phase duration (λ), initial cell concentration (*y*_0_), coefficient of determination (R^2^), and standard error (SE) of fit.

(1) $$Y=Y_{0}+\mu A\left(t\right)-ln(1+\frac{\exp[\mu A(t)]+1}{\exp\left(ymax-y_{0}\right)})$$

*y* = cell number (log CFU/g).

*y*_0_ = log initial number of cells (log CFU/g).

A = difference between initial and final cell numbers (CFU/g).

*t* = time.

*μ* = maximum specific growth rate.

λ = lag phase duration.

*ymax* = maximum cell number.

### 2.4. Statistical Analysis

Data were analyzed using SPSS Statistics 26.0 (IBM, Armonk, NY, USA) and Microsoft Excel 2024. To assess the effectiveness of each disinfectant type and at varying concentrations, one-way analysis of variance was performed, and statistical significance was set at a *p*-value of 0.05.

## 3. Results

### 3.1. Microbial Contamination Levels of General Produce and Fresh-Cut Vegetables

Table 2 shows the levels of TMC, *E. coli*, and *L. monocytogenes* in these five fresh-cut salad products and four types of raw produce. The average level of TMC of the five products was 2.00 log CFU/g. By product type, the chicken Caesar salad product showed no detectable TMC, whereas the baby leafy vegetable and sprout salad exhibited the highest TMC of 4.26 log CFU/g, followed by 3.46 log CFU/g for the lettuce and romaine salad, 1.26 log CFU/g for the lettuce and cabbage salad, and 1.01 log CFU/g for the lettuce and red cabbage salad. For the four types of raw produce, the average level of TMC was 2.98 log CFU/g, with the highest level observed in crown daisy (4.14 log CFU/g), followed by carrots (2.74 log CFU/g), lettuce (2.57 log CFU/g), and cucumbers (2.47 log CFU/g).

For *E. coli*, the mean contamination level in the fresh-cut salad products was 2.03 log CFU/g. The highest levels of contamination were observed in the baby leaf and sprout salad (3.90 log CFU/g), whereas *E. coli* was not detected in the chicken salad. The average contamination level of *E. coli* in general produce was 1.53 log CFU/g. No contamination was detected in lettuce and carrots, while higher levels were detected in crown daisy (3.38 log CFU/g) and cucumbers (2.47 log CFU/g).

The average contamination level of *L. monocytogenes* in the fresh-cut vegetable salad products was 1.80 log CFU/g, with the highest levels observed in the baby leaf and sprout salad (3.84 log CFU/g), followed by the lettuce and romaine salad (2.20 log CFU/g), the lettuce and cabbage salad (2.22 log CFU/g), and the lettuce and red cabbage salad (0.75 log CFU/g). In the case of general produce, the mean contamination level of *L. monocytogenes* was 2.22 log CFU/g. Although crown daisies showed the maximum levels of *L. monocytogenes* (3.85 log CFU/g), followed by cucumbers (2.71 log CFU/g) and carrots (2.32 log CFU/g), none was detected in lettuce.

### 3.2. Microbial Reduction Capability According to Disinfection Method

Results of the levels of TMC, *E. coli*, and *L. monocytogenes* on green onions and chives after the disinfection treatments are presented in Table 3 and Table 4, respectively. Significant reductions in TMC, *E. coli*, and *L. monocytogenes* were observed in green onion samples after disinfection treatment. The TMCs of unwashed green onions, those washed three times with running water, those treated with 100 ppm NaClO in electrolyzed water, and those immersed in the commercial chemical disinfectant (Yuhan Clorox), were 4.17, 1.24, 1.38 and 1.66 log CFU/g, respectively. Moreover, TMC on the green onions was not detected in the other electrolyzed water treatment groups (acidic 30 ppm HOCl, acidic 60 ppm HOCl, and alkaline 200 ppm NaClO), confirming the significance of disinfecting effect (*p* < 0.05). These results suggest that washing three times with running water can reduce the levels of TMC on green onions by 2.9 log CFU/g. The acidic 30 ppm HOCl, acidic 60 ppm HOCl, and alkaline 200 ppm NaClO reduced the TMC by more than 4 log CFU/g.

The mean TMC of the non-washed chives was 6.98 log CFU/g (Table 4), which was approximately 2 log units higher than that of green onions. The reduction effects of TMC by each treatment showed in the following order: 4.80 log CFU/g with acidic 60 ppm HOCl electrolyzed water, 4.74 log CFU/g with alkaline 100 ppm NaClO electrolyzed water, 4.69 log CFU/g with alkaline 200 ppm NaClO electrolyzed water, 4.42 log CFU/g with acidic 30 ppm HOCl electrolyzed water, 4.03 log CFU/g with 3 time water washing, and 3.78 log CFU/g with 100 ppm NaClO chemical solution. The results indicated a significantly higher disinfection against TMC by 60 ppm HOCl, as well as 100 and 200 ppm NaClO electrolyzed water, than the other intervention methods.

The contamination levels of *E. coli* and *L. monocytogenes* in unwashed raw green onions were 2.60 and 2.78 log CFU/g, respectively (Table 4), whereas neither pathogen was detected in any of experiment groups. Conversely, chives showed higher contamination levels of *E. coli* and *L. monocytogenes* than green onions, indicating values of 3.28 and 3.15 log CFU/g, respectively (Table 4). Furthermore, no *E. coli* and *L. monocytogenes* were observed in the electrolyzed water groups generated by the device, confirming its disinfection efficacy.

### 3.3. Predictive Modeling of L. monocytogenes Growth on Chive Under Different Storage Conditions

**Figure 1:**
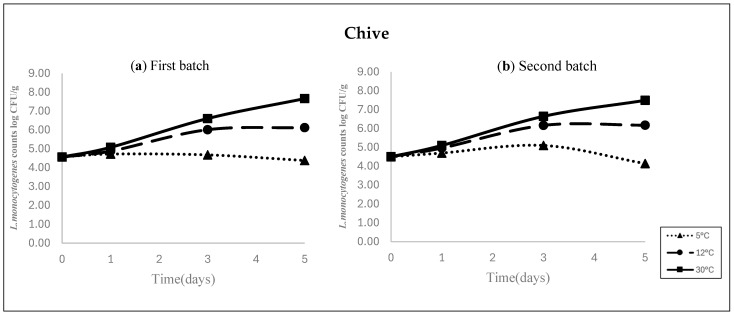
Growth curves of *L. monocytogenes* inoculated on *buchu* (Asian chives) stored at 5 °C, 12 °C, and 30 °C. Growth was assessed in two independent batches for each temperature condition: Batch 1 (**a**) and Batch 2 (**b**). Data are presented as mean values.

The present study applied the Baranyi and Roberts model [18] to describe the temperature-dependent growth of *L. monocytogenes*, and the fitted growth curves are shown in Figure 1. Table 5 shows the estimated model parameters, including the initial cell concentration (y_0_), maximum growth rate (μ), maximum cell number (Vmax), and lag phase duration (LPD), along with the model performance indicators such as the coefficient of determination (R^2^) and the SE of the fit. As shown in Figure 1, *L. monocytogenes* growth was monitored for 5 days at 5 °C, 12 °C, and 30 °C. In the inoculated samples, the initial populations were 4.57 and 4.51 log CFU/g for Batch 1 and 2, respectively. The R^2^ values for the inoculated samples ranged from 0.96 to 0.99 across all the tested temperatures in Batch 1 (12 °C and 30 °C) and Batch 2 (12 °C and 30 °C), indicating a high degree of model fit to the experimental data. Moreover, the growth rate of *L. monocytogenes* on chives increased approximately 15-fold from 5 °C to 30 °C. Due to the relatively high initial inoculation levels in the treated groups, the lag phase was not apparent. The maximum population densities during the stationary phase with the inoculated samples were as follows: Batch 1, 6.15 log CFU/g at 12 °C and 7.83 log CFU/g at 30 °C, and Batch 2, 6.21 log CFU/g at 12 °C and 7.55 log CFU/g at 30 °C. Such densities at 5 °C could not be calculated because of no growth of *L. monocytogenes.* Regarding “µ”, the values at 5 °C were negative in both batches (–0.002 log CFU/h). At 12 °C, the μ values were 0.021 and 0.025 log CFU/h, while at 30 °C, they increased to 0.029 and 0.030 log CFU/h for Batch 1 and 2, respectively. These findings confirm that *L. monocytogenes* were unable to grow at 5 °C, whereas it proliferated prominently at 12 °C, suggesting that storage at a temperature below 5 °C effectively controls *L. monocytogenes* growth on chives, not 12 °C.

## 4. Discussion

With the increase in single-person households and growing interest in healthy eating habits, the demand for ready-to-eat fresh-cut produce without any pre-preparation has increased [19]. However, such products are susceptible to contamination by foodborne pathogens and food-poisoning risks are elevated, since they are consumed without heating. The CDC estimated the average number of foodborne illnesses, hospitalizations, and deaths in the United States in 2019 and reported invasive *L. monocytogenes* infections totaled 1250 cases per year, comprising 1050 non-pregnancy-associated and 198 (106 mothers and 92 infants) pregnancy-associated illnesses. A total of 1068 hospitalizations was caused by invasive *L. monocytogenes*, of which 920 were nonpregnancy, and 148 (74 mothers, 74 infants) were pregnancy-associated. In addition, 172 deaths were reported, of which 166 were nonpregnancy, and 6 (all infants) were pregnancy-associated deaths [2]. Therefore, microbiological control measures specific to fresh-cut vegetables during production or delivery should be implemented, especially for ensuring safety under mass-production foodservice settings [20,21]. According to the “Common Standards and Specifications for Foods” in the Korean Food Code [14], the criteria for *L. monocytogenes* applicable to processed foods that are sterilized, pasteurized or intended for consumption without further processing or heating, require its complete absence (n = 5, c = 0, m = 0 per 25 g). Hygiene guidelines for vegetables and fruits without a heating process in institutional foodservice settings require disinfection process using a prescribed method [20,21].

In the present study, the microbiological quality of crown daisies in general produce, as well as the baby leaf and sprout salad among the fresh-cut vegetables, needs improvement. Although no legal standards on TMCs for raw-vegetable produce or fresh-cut vegetable products have been established in Korea [14], Solberg et al. [22] have recommended a TMC criterion of less than 5 log CFU/g to ensure the microbiological quality for ready-to-eat products. Based on the criteria, all fresh-cut salad products tested in the study had TMCs within the acceptable range, indicating a lower food-poisoning risk if washed properly. However, even though the confirmation tests for *E. coli* and *L. monocytogenes* were not conducted, compared with other vegetables, fresh-cut vegetables such as the baby leaf and sprout salad and crown daisy require continuous quality management during the distribution. In a Brazilian study, *L. monocytogenes* was detected in 11.2% of food samples, with 71 positives among 635 food samples. By food types, the highest prevalence of *L. monocytogenes* was detected with frozen foods (38%, 17 samples), cheese (10%, 4 samples), ready-to-eat (9%, 42 samples, sandwiches, vegetable salad, etc.), and deli meat (7%, 8 samples) [23].

This present study confirmed that the effectiveness of chlorine-based disinfectants depends on their concentration, supporting their use in the hygiene management of salad dishes. Recently, various disinfectants have been widely used to prevent food poisoning and to ensure food safety; however, a gap has been observed between their intended use in foodservice settings and consumer awareness [24]. The KMFDS has approved seven types of food disinfectants to ensure food quality control: hydrogen peroxide (H_2_O_2_), ozone water, chlorine dioxide (ClO_2_), NaClO, HOCl, calcium hypochlorite (Ca[ClO]_2_), and peracetic acid (CH_3_COOOH) [10,11]. Among them, chlorine-based disinfectants have been widely used in the sanitization of fresh vegetables for institutional foodservice meals and fresh-cut vegetables, as well as eco-friendly disinfectants in various fields, such as medicine and agriculture. In particular, HOCl and NaClO can be easily produced through electrolysis of tap water containing chloride ions. Furthermore, the United States Environmental Protection Agency officially approved electrolysis water generators as “disinfectant generators” in 1998. A study conducted from 25 October to 7 November 2017, on the use of disinfectants in 109 institutional foodservice settings in Korea reported that 16.5% used disinfectant-water generation devices as well as HOCl water or NaClO for sanitizing vegetables and fruits [7]. This suggests that the use of the disinfectant-water generation devices is relatively low, and most institutional foodservices dilute Yuhan Clorox to obtain a 100 ppm disinfectant solution for sanitizing vegetables. As this study confirmed its disinfecting capacity, the use of this device to produce water for the disinfection of vegetables and fruits is expected to make the disinfection process more accurate and convenient. However, a total of 13 cases related to defects in electrolyzed water generators were reported to the consumer safety monitoring system from January 2019 to September 2020: product defects (four cases), safety concerns (four cases), lack of effectiveness (three cases), and odor issues (two cases), suggesting that consumers may still have concerns about the safety of electrolyzed water generators.

Concerns about the safety of electrolysis water generators were associated with the pH of the generated water. Tashiro et al.’s clinical study suggested that electrolyzed water with a pH over 9.8 can cause growth retardation, inhibition of nutrient absorption, and excessive growth of pathogenic bacteria [25]. Stankevič et al. [26] also confirmed through animal experiments that electrolyte water with a higher pH can induce hypercalcemia and kidney function disorders. Furthermore, food hygiene standards in Korea recommend that sanitized vegetables and fruits should be immersed in clean water or rinsed with running water 2-3 times after disinfection to remove any residual disinfectants [11]. The pH levels of the electrolyzed water used in this study were 8.1 at 100 ppm NaClO and 8.8 at 200 ppm NaCl, indicating that they were below the pH standard suggested in previous studies [11,12,13,14]. In the case of HOCl, given that the pH level ranged from 3.2 to 5.6, it is safer from skin irritation and toxicity concerns. Moreover, since HOCl is a highly volatile chlorine compound that can be easily dissolved in the air, there is little chance that this compound will remain in food ingredients [11].

The United States Food and Drug Administration also generally recognized HOCl as a safe material. Thus, washing with electrolyzed water containing NaOCl and HOCl can be an effective sterilization method for controlling the pathogen microorganisms in vegetables. Thus, using this device for vegetable and fruit disinfection will facilitate the disinfection management process more accurately and conveniently. The quantitative microbial risk assessment (QMRA) for *E. coli* O157:H7 in fresh-cut lettuce also pointed out that a chlorine intervention strategy could reduce the possibility of contamination-associated illnesses by 12.7-fold compared with the mean predicted number of 2160 illnesses per year [27].

The microbiological safety of fresh-cut vegetables has been a topic of concern due to raw ingredients that have been commonly served without heating. Careless practices during production, storage, and distribution can lead to *L. monocytogenes*-induced food poisoning. It is a gram-positive, non-spore-forming pathogen and psychrotrophic microorganism that can survive at refrigeration temperatures ranging from 4 to 15 °C [28,29]. Diced celery was recognized as the cause of a listeriosis outbreak in Texas [13,29], and cucumbers contaminated with *E. coli O157:H7* caused a disease outbreak in Germany [30]. In addition, predictive models for the growth of *L. monocytogenes* have been developed using vegetables and fruit such as fresh-cut cantaloupes [31,32], onions [5,13], and cucumbers contaminated with *L. monocytogenes* [33,34]. The growth of *L. monocytogenes* on fresh-cut mixed salad at different pre-growth temperatures (4, 21, and 37 °C) was fitted using the Baranyi–Roberts model [13]. The growth kinetics of *L. monocytogenes* on cooked vegetables, such as carrots, corn, onions, green bell peppers, and potatoes, were tested. The vegetables were cooked till an internal temperature of 90 °C, cooled to 5 °C, inoculated with *L. monocytogenes*, and stored at different storage temperatures (i.e., 5, 10, or 25 °C) for 7 days. *L. monocytogenes* survived on all the vegetables at a storage temperature of 5 °C. At 10 °C, it proliferated on all the vegetables except peppers. At 25 °C, the highest growth rates were observed on carrots (5.55 log CFU/g/day), followed by corn (4.02 log CFU/g/day), peppers (3.27 log CFU/g/day), onions (1.39 log CFU/g/day), and potatoes (0.91 log CFU/g/day). Unlike the previous study, our study showed the growth rate of *L. monocytogenes* on chives was negative at 5 °C, and even very low at 35 °C.

This result can be attributed to the high initial bacterial concentration of 4 log CFU/g, the lower nutritional content of leeks than protein-rich foods, and a lower pH due to acidic compounds produced during microbial growth. The growth rate of *L. monocytogenes* in produce is affected by the food surface structure, nutritional composition, water activity, storage conditions such as temperature and relative humidity, and packaging conditions [7]. Other studies also state that extracts of chive (*Allium tuberosum*) have a retardation effect of Kimchi fermentation, particularly by strongly inhibiting the growth of *Pediococcus*, *Lactobacillus*, and *Leuconostoc* spp. [35]. Fresh chive extracts have antimicrobial activities against *S. aureus* and *E. coli*. The active compound responsible likely has a small molecule that is lower than 12,000 [36]. Future studies should consider measuring and incorporating nutrient levels, pH, and salt concentration of foods to develop accurate growth prediction model for *L. monocytogenes*.

## 5. Conclusions

This study provides significant contributions as it assessed the disinfectant efficacy of various disinfectants on raw vegetables and by developing growth models for *L. monocytogenes* on chives under different storage temperatures for 7 days. Disinfection experiments with chives and green onions revealed high efficacy of electrolyzed water containing alkaline 100 ppm and 200 ppm NaOCl as well as acidic 30 ppm and 60 ppm HOCl against TMC, *E. coli*, and *L. monocytogenes*. Furthermore, in the *L. monocytogenes* inoculation experiment in chives, growth rates at 5 °C, 12 °C, and 30 °C were –0.002, 0.023, and 0.03 log CFU/g/h, respectively. This may be due to the antioxidant effect of chive, and future studies are needed to identify the relations. These results provide valuable evidence to support the development of food safety and the establishment of standards for microbiological quality control in the catering and the broader food industry.

## Figures and Tables

**Table 1:** Composition of fresh-cut vegetable salad samples (n = 5).

Sample	Ingredient Composition	Manufacturer
Chicken Caesar salad	Lettuce, paprika, chicken breast, quail egg, chicory, red beet, tomato, romaine, and crouton	A
Lettuce and romaine salad	Lettuce, romaine, red mustard, red chard, and cabbage	B
Lettuce and cabbage salad	Lettuce, cabbage, red cabbage, romaine, frill ice lettuce, butterhead, Batavia, and radicchio	C
Lettuce and red cabbage salad	Lettuce, red cabbage, cabbage, chicory, and red beet	D
Young leafy vegetables and sprouts salad	Young leafy vegetables, sprouts, red radish sprouts, and red radish	B

The number of fresh-cut vegetable product samples was five. Samples were taken twice, during winter and spring.

**Table 2:** Microbial contamination levels in fresh-cut vegetables and general agricultural produce (Mean ± SD).

Classification	Sample	Contamination Levels (log CFU/g)		
		TMC	Presumptive *E. coli* ^(^^1)^	Presumptive *L. monocytogenes*
Fresh-cut vegetable	Chicken Caesar salad	ND	ND	ND
	Lettuce and romaine salad	3.46 ± 0.56	3.15 ± 0.07	2.20 ± 0.13
	Lettuce and cabbage salad	1.26 ± 1.78	2.16 ± 0.51	2.22 ± 0.44
	Lettuce and red cabbage salad	1.01 ± 1.42	0.95 ± 1.34	0.75 ± 1.06
	Baby leafy vegetables and sprouts salad	4.26 ± 0.21	3.90 ± 0.28	3.84 ± 0.57
	Average	2.00 ± 1.79	2.03 ± 1.59	1.80 ± 1.49
Agricultural produce (no cut)	Lettuce	2.57 ± 0.41	ND	ND
	Crown daisy	4.14 ± 0.62	3.38 ± 0.53	3.85 ± 0.59
	Cucumber	2.47 ± 0.39	2.74 ± 0.44	2.71 ± 0.43
	Carrot	2.74 ± 0.44	ND	2.32 ± 0.37
	Average	2.98 ± 0.78	1.53 ± 1.78	2.22 ± 1.62

^(1)^ The term “presumptive” is used because further confirmation tests were not performed for *E. coli* and *L. monocytogenes*; ND: not detected, <4 CFU/g.

**Table 3:** Changes in the levels of TMC, *E. coli*, and *L. monocytogenes* in green onions according to disinfection types (Mean ± SD).

Bacteria (log CFU/g)	Unwashed	Water Washing ^(2)^	100 ppm (NaCIO) ^(3)^	Electrolyzed Water ^(4)^				*F*	*p*-Value
				30 ppm	60 ppm	100 ppm	200 ppm		
				(HOCl)	(HOCl)	(NaClO)	(NaClO)		
TMC	4.17 ± 4.05 ^b (1)^	1.24 ± 1.45 ^a^	1.66 ± 1.73 ^a^	ND ^a (6)^	ND ^a^	1.38 ± 1.60 ^a^	ND ^a^	5.68	0.001
Presumptive *E. coli* ^(5)^	2.60 ± 2.54 ^b^	ND ^a^	ND ^a^	ND ^a^	ND ^a^	ND ^a^	ND ^a^	5.91	0.002
Presumptive *L. monocytogenes* ^(5)^	2.78 ± 2.15 ^b^	ND ^a^	ND ^a^	ND	ND ^a^	ND ^a^	ND ^a^	71.8	<0.001

^(1)^ Different letters within a row indicate significant differences at *p* = 0.05 according to the Duncan test; ^(2)^ washed three times with running warm water; ^(3)^ chemical solution; ^(4)^ electrolyzed water generated using the device; ^(5)^ the term “presumptive” is used because further confirmation tests were not conducted for *E. coli* and *L. monocytogenes*; ^(6)^ ND: not detected.

**Table 4:** Changes in the levels of TMC, *E. coli*, and *L. monocytogenes* in chives according to disinfection types (Mean ± SD).

Bacteria (log CFU/g)	Unwashed	Water Washing ^(2)^	100 ppm (NaCIO) ^(3)^	Electrolyzed Water ^(4)^				*F*	*p*-Value
				30 ppm	60 ppm	100 ppm	200 ppm		
				(HOCl)	(HOCl)	(NaClO)	(NaClO)		
TMC	6.98 ± 2.73 ^b (1)^	2.96 ± 2.73 ^a^	3.20 ± 2.36 ^a^	3.20 ± 2.36 ^a^	2.18 ± 1.42 ^a^	2.24 ± 1.93 ^a^	2.29 ± 1.96 ^a^	26.69	<0.001
Presumptive *E. coli* ^(5)^	3.28 ± 2.99 ^b^	ND ^a (6)^	ND ^a^	ND ^a^	ND ^a^	ND ^a^	ND ^a^	13.88	<0.001
Presumptive *L. monocytogenes* ^(5)^	3.15 ± 2.93 ^b^	ND ^a^	ND ^a^	ND ^a^	ND ^a^	ND ^a^	ND ^a^	11.07	<0.001

^(1)^ Different letters within a row indicate significant difference at *p* = 0.05 according to the Duncan test; ^(2)^ washed three times with running warm water; ^(3)^ chemical solution; ^(4)^ electrolyzed water generated using the device; ^(5)^ the term “presumptive” is used because further confirmation tests were not conducted for *E. coli* and *L. monocytogenes*; ^(6)^ ND: not detected.

**Table 5:** Growth curves of *L. monocytogenes* inoculated on chives at different storage temperatures (Mean ± SD).

Batch	Temperature (°C)	Model	μ (log CFU/g/h)	*y*_0_ (log CFU/g)	*ymax* (log CFU/g)	λ (h)	R^2^	SE of Fit
1	5	Baranyi and Roberts	−0.002 ± 0.002	4.682 ± 0.11	NA	NA ^(1)^	0.060	0.143
	12	Baranyi and Roberts	0.021 ± 0.004	4.494 ± 0.134	6.145 ± 0.155	NA	0.96	0.154
	30	Baranyi and Roberts	0.029 ± 0.003	4.497 ± 0.117	7.830 ± 0.317	NA	0.99	0.138
2	5	Baranyi and Roberts	−0.002 ± 0.005	4.738 ± 0.363	NA	NA	NA	0.472
	12	Baranyi and Roberts	0.025 ± 0.004	4.458 ± 0.11	6.210 ± 0.118	NA	0.98	0.121
	30	Baranyi and Roberts	0.030 ± 0.002	4.456 ± 0.0875	7.551 ± 0.132	NA	0.994	0.103

^(1)^ NA: not applicable; μ: maximum growth rate; *y*_0_: the initial cell concentration; *ymax:* maximum cell number; λ: lag phase duration; R^2^: the model performance indicators (the coefficient of determination).

## Data Availability

The original contributions presented in this study are included in the article. Further inquiries can be directed to the corresponding author.

## References

[1] Hwang H.S., Jeong J.H., Kwon Y.H., Byun Y.J., Park J.Y., Yun H.C. (2024). A study of microbial contamination in fresh-cut and ready-to-eat foods purchased from online markets. J. Food Hyg. Saf..

[2] Walter E.J.S., Cui Z., Tierney R., Griffin P.M., Hoekstra R.M., Payne D.C., Rose E.B., Devine C., Namwase A.S., Mirza S.A. (2025). Foodborne illness acquired in the United States-Major pathogens, 2019. Emerg. Infect. Dis..

[3] Centers for Disease Control and Prevention (CDC) Attribution of Foodborne Illness. https://www.cdc.gov/food-safety/php/data-research/foodborne-illness-sources/index.html.

[4] National Institute of Food and Drug Safety Evaluation (NIFDSE) (2024). Risk Assessment of Listeria Monocytogenes in Vegetables and Processed Vegetable Products.

[5] Kim G.H., Lim J.Y., Kim Y.H., Yang S.Y., Yoon K.S. (2020). Development of predictive models of *Listeria monocytogenes* in fresh-cut fruits and vegetables. J. Food Hyg. Saf..

[6] Ju S.Y., Ko J.J., Yoon H.S., Seon S.J., Yoon Y.R., Lee D.I., Kim S.Y., Chang H.J. (2017). Does electrolyzed water have different sanitizing effects than sodium hypochlorite on different vegetable types?. Br. Food J..

[7] Bae H.J. (2018). A study on improvement of usage standards for food sanitizers (chlorine dioxide, sodium hypochlorite). Final Research Report.

[8] Marshall K.E., Hexeme A., Seelman S.L., Fatica M.K., Blessington T., Hajmeer M., Kisselburgh H., Atkinson R., Hill K., Sharma D. (2020). Lessons learned from a decade of investigations of Shiga toxin–producing Escherichia coli outbreaks linked to leafy greens, United States and Canada. Emerg. Infect. Dis..

[9] Franz E., Tromp S.O., Rijgersberg H., van der Fels-Klerx H.J. (2010). Quantitative microbial risk assessment for *Escherichia coli* O157:H7, *Salmonella,* and *Listeria monocytogenes* in leafy green vegetables consumed at salad bars. J. Food Prot..

[10] Ministry of Food and Drug Safety (MFDS) (2024). Safe Use of Food Sanitizers: Approved Seven Food-Grade Disinfectants including Hydrogen Peroxide, Ozone Water, Peracetic Acid, Sodium Hypochlorite, Calcium Hypochlorite, Hypochlorous Acid, and Chlorine Dioxide. Food Safety Knowledge Board.

[11] Ministry of Food and Drug Safety (MFDS) (2022). Field Guidelines for Food Sanitizers; Revised Edition.

[12] Marik C.M., Zuchel J., Schaffner D.W., Strawn L.K. (2020). Growth and survival of *Listeria monocytogenes* on intact fruit and vegetable surfaces during postharvest handling: A systematic literature review. J. Food Prot..

[13] Salazar J.K., Fay M.L., Khouja B.A., Mate M., Zhou X., Lingareddygari P., Liggans G. (2024). Dynamics of *Listeria monocytogenes* and *Salmonella enterica* on cooked vegetables during storage. J. Food Prot..

[14] Ministry of Food and Drug Safety (MFDS) Korean Food Code. https://www.foodsafetykorea.go.kr/foodcode/01_03.jsp.

[15] Leininger D.J., Roberson J.R., Elvinger F. (2001). Use of eosin methylene blue agar to differentiate *Escherichia coli* from other gram-negative mastitis pathogens. J. Vet. Diagn. Investig..

[16] KisanBio Co., Ltd. MB Cell Product Information. https://www.kisanbio.com.

[17] Jee D., Ha J. (2021). Synergistic interaction of tap water-based neutral electrolyzed water combined with UVA irradiation to enhance microbial inactivation on stainless steel. Food Res. Int..

[18] Baranyi J., Roberts T.A. (1994). A dynamic approach to predicting bacteria growth in food. Int. J. Food Microbiol..

[19] Ha J.Y., Lim S.H. (2022). Analysis of salad purchaser types and purchasing behaviors through social network analysis. J. Korean Soc. Qual. Manag..

[20] Ministry of Education (2021). School Meal Hygiene Management Guidelines. Health Promotion Center. https://www.schoolkeepa.or.kr/cop/bbs/selectArticleDetail.do?nttId=431&bbsId=BBSMSTR_000000000021.

[21] Ministry of Food and Drug Safety (2022). Guidelines for the Children’s Meal Management Support Center. https://dietary4u.mfds.go.kr/board.es?mid=a61303000000&bid=0256&act=view&list_no=75288&tag=&nPage=1.

[22] Solberg M., Buckalew J.J., Chen C.M., Schaffner D.W., O’neil K., McDowell J., Post L.S., Boderch M. (1990). Microbiological safety assurance system for foodservice facilities. Agricultural and Food Sciences, Environmental Science Food Technol..

[23] Braga V., Vázquez S., Vico V., Pastorino V., Motab M.I., Legnani M., Schelotto F., Lancibidad G., Varela G. (2017). Prevalence and serotype distribution of *Listeria monocytogenes* isolated from foods in Montevideo-Uruguay. Braz. J. Microbiol..

[24] Korea Consumer Agency (2021). Safety survey on electrolyzed water generators. Safety Report.

[25] Tashiro H., Kitahora T., Sumiyoshi K., Fujiyama Y., Bamba T. Effect of functional water on gastrointestinal physiology. Proceedings of the Second Function Water Symposium ’95.

[26] Stankevič J., Audickaitė A., Šilovė S., Šimčikas V., Cesiulis H., Skujienė G., Bukelskienė V., Žalgevičienė V., Tutkuvienė J. (2020). Effect of ionised (electrolysed) water on the rat embryo development. J. Environ. Eng. Landsc. Manag..

[27] Pang H., Lambertini E., Buchanan R.L., Schaffner D.W., Prandhan A.K. (2017). Quantitative microbial risk assessment for *Escherichia coli* O157:H7 in fresh-cut lettuce. J. Food Proct..

[28] Manyi-Loh C.E., Lues R. (2025). *Listeria monocytogenes* and listeriosis: The global enigma. Foods.

[29] Gaul L.K., Farag N.H., Shim T., Kingsley M.A., Silk B.J., Hyytia-Trees E.H. (2013). Hospital-acquired listeriosis outbreak caused by contaminated diced celery in Texas, 2010. Clin. Infect. Dis..

[30] Tuffs A. (2011). Outbreak of E. coli in Germany is linked to cucumbers from Spain. Br. Med. J..

[31] Huang J., Luo Y., Nou X. (2015). Growth of *Salmonella enterica* and *Listeria monocytogenes* on fresh-cut cantaloupe under different temperature abuse scenarios. J. Food Prot..

[32] Huang J., Luo Y., Zhou B., Zheng J., Nou X. (2019). Growth and survival of Salmonella enterica and *Listeria monocytogenes* on fresh-cut produce and their juice extracts: Impacts and interactions of food matrices and temperature abuse conditions. Food Control..

[33] Jeon J.H., Roh J.H., Lee C.L., Kim G.H., Lee J.Y., Yoon K.S. (2022). Microbial qualities of parasites and foodborne pathogens in ready-to-eat (RTE) fresh-cut produces at the on/offline markets. J. Food Hyg. Saf..

[34] Feng K., Sarengaowa, Ma J., Hu W. (2024). Modelling the growth of *Listeria monocytogenes* on fresh-cut cucumbers at various storage temperatures. Horticulturae.

[35] Kim S., Park K. (1995). Retardation of Kimchi fermentation by the extracts of *Allium tuberosum* and growth inhibition of related microorganisms. Korean J. Food Sci. Technol..

[36] Lee M.K., Lee J., Park I. (2001). Growth retardation of *Escherichia coli* and *Staphylococcus aureus* by leek extract. J. Korean Sci. Food Sci. Nutr..

